# Genetic diversity of SARS-CoV-2 and clinical, epidemiological characteristics of COVID-19 patients in Hanoi, Vietnam

**DOI:** 10.1371/journal.pone.0242537

**Published:** 2020-11-17

**Authors:** Tam Thi Nguyen, Thach Ngoc Pham, Trang Dinh Van, Trang Thu Nguyen, Diep Thi Ngoc Nguyen, Hoa Nguyen Minh Le, John-Sebastian Eden, Rebecca J. Rockett, Thuong Thi Hong Nguyen, Bich Thi Ngoc Vu, Giang Van Tran, Tan Van Le, Dominic E. Dwyer, H. Rogier van Doorn, on behalf of the OUCRU COVID-19 Research Group

**Affiliations:** 1 Oxford University Clinical Research Unit (OUCRU), Hanoi, Vietnam; 2 National Hospital for Tropical Diseases, Hanoi, Vietnam; 3 The University of Sydney, Marie Bashir Institute for Infectious Disease and Biosecurity, Westmead, New South Wales, Australia; 4 Westmead Institute for Medical Research, Centre for Virus Research, Westmead, New South Wales, Australia; 5 Centre for Infectious Diseases and Microbiology – Public Health, Institute of Clinical Pathology and Medical Research, Westmead Hospital, Westmead, New South Wales, Australia; 6 Department of Infectious Diseases, Hanoi Medical University, Hanoi, Vietnam; 7 Department of Virology and Parasitology, National Hospital for Tropical Diseases, Hanoi, Vietnam; 8 Oxford University Clinical Research Unit, Ho Chi Minh city, Vietnam; 9 Centre for Infectious Diseases and Microbiology Laboratory Services, New South Wales Health Pathology – Institute of Clinical Pathology and Medical Research, Westmead, New South Wales, Australia; 10 Nuffield Department of Clinical Medicine, University of Oxford, Oxford, United Kingdom; Institute of Human Virology, UNITED STATES

## Abstract

A second cluster of COVID-19 cases imported from Europe occured in Vietnam from early March 2020. We describe 44 SARS-CoV-2 RT-PCR positive patients (cycle threshold value <30) admitted to the National Hospital for Tropical Diseases in Hanoi between March 6 and April 15 2020. Whole SARS-CoV-2 genomes from these patients were sequenced using Illumina Miseq and analysed for common genetic variants and relationships to local and globally circulating strains. Results showed that 32 cases were Vietnamese with a median age of 37 years (range 15–74 years), and 23 were male. Most cases were acquired outside Vietnam, mainly from the UK (n = 15), other European countries (n = 14), Russia (n = 6) and countries in Asia (n = 3). No cases had travelled from China. Forty-one cases had symptoms at admission, typically dry cough (n = 36), fever (n = 20), sore throat (n = 14) and diarrhoea (n = 12). Hospitalisation was long with a median of 25 days, most commonly from 20–29 days. All SARS-CoV-2 genomes were similar (92–100% sequence homology) to the reference sequence Wuhan_1 (NC_045512), and 32 strains belonged to the B.1.1 lineage. The three most common variants were linked, and included C3037T, C14408T (nsp12: P323L) and A23403G (S: D614G) mutations. This group of mutations often accompanied variant C241T (39/44 genomes) or GGG 28881..28883 AAC (33/44 genomes). The prevalence of the former reflected probable European origin of viruses, and the transition D614G was dominant in Vietnam. New variants were identified; however, none could be associated with disease severity.

## Introduction

Coronavirus Disease 2019 (COVID-19) is caused by the severe acute respiratory syndrome coronavirus 2 (SARS-CoV-2) and has spread to 218 countries and territories worldwide, leading to more than 42,500,000 confirmed cases with 1,147,301 deaths by October 25 2020 [1]. The number of deaths due to COVID-19 has increased significantly since April 2020, with between 3000 to 6000 deaths daily [2].

SARS-CoV-2 belongs to family *Coronaviridae*, genus Betacoronavirus and subgenus Sarbecovirus, and is one of seven coronaviruses causing human disease—NL63, 229E, OC43, HKU1, SARS-CoV and Middle East Respiratory Syndrome CoV (MERS-CoV) [3]. The SARS-CoV-2 genome has about 89% nucleotide sequence similarity with SARS-like CoVs (bat SL-CoVZC45, SARS-CoV Tor2) and is divided into two untranslated terminal regions (UTR) and 14 open reading frames (ORFs). The structural ORFs consist of Spike (S), Envelope (E), Membrane (M) and Nucleocapsid (N) proteins, while other regions code for non-structural or accessory proteins [4].

On January 23 2020, the Ministry of Health in Vietnam reported the first imported case of SARS-CoV-2 infection, someone who had recently travelled from Wuhan in Hubei Province, China. A total of 16 cases were recorded in this first cluster, but more SARS-CoV-2 importation and transmission in Vietnam happened from March 2020 onward, when residents and tourists entered Vietnam from Europe, USA and elsewhere in Asia [5]. Notably, local community transmission was successfully suppressed for 99 days from the April 15 to July 24 2020 [5]. The cases recently detected in Da Nang have triggered the third cluster of COVID-19 in Vietnam, increasing local and hospital acquired transmission and deaths. There are a number of reports describing the clinical, epidemiological and genetic features of COVID-19 cases and SARS-CoV-2 in Vietnam, although whole genome sequencing (WGS) data is limited [6–18]. Here, we report the clinical, epidemiological and WGS features and correlations of 44 patients with COVID-19 in Vietnam.

## Materials and methods

### Samples collection and molecular diagnosis

Nasopharyngeal and oropharyngeal swabs of all suspected COVID-19 cases quarantined or hospitalised/isolated at the National Hospital for Tropical Diseases (NHTD) were collected using sterile cotton buds and stored in in-house produced viral transport medium. Samples were tested within 24 hours of collection. Total RNA was extracted with the QIAamp viral RNA mini kit (Qiagen, Hilden, Germany) and tested with a real-time reverse transcription PCR (real-time RT-PCR) to check for the presence of the *E* gene (112bp) and RNA-dependent RNA polymerase (*RdRp*) gene (99bp) according to the World Health Organization’s protocol using SuperScript III Platinum One-Step qRT-PCR kit (Invitrogen, Carlsbad, CA USA) and E-Sarbeco, RdRp primers and probes (Tib Molbiol, Berlin, Germany) [19]. Positive samples with cycle threshold (Ct) values <30 for both genes were stored at -80°C for sequencing.

### cDNA synthesis, PCR amplification and whole genome sequencing

We obtained 44 real-time RT PCR positive samples (Ct < 30) diagnosed from March 6 to April 15 2020, and SARS-CoV-2 WGS was performed as described elsewhere [20, 21]. Briefly, RNA was converted into cDNA, then amplified into 14 PCR products spanning the SARS-CoV-2 reference genome (Accession: MN 908947-Wuhan-Hu-1). PCR products (about 2.5kb) were checked by electrophoresis using a 1% agarose gel running at 110V for 30 minutes. PCR fragments of each sample were then pooled and purified by AMPure XP beads (Beckman Coulter, USA).

Library preparation used the Nextera XT Library preparation kit (Illumina, USA), and sequencing was performed on an Illumina Miseq platform with the 300 cycle v2 or 300 cycle micro v2 kit (Illumina, USA) following manufacturer’s instructions.

### Collection of clinical and epidemiological information

Real-time RT PCR positive patients were admitted to the NHTD, the first-line national hospital for COVID-19 treatment in northern Vietnam. Medical staff recorded patient demographics, travel history and contacts within the previous 14 days. Admission signs, symptoms and health history were obtained by direct patient communication, and followed up during hospitalisation. Treatments, chest radiography or computed tomography (CT) and clinical outcomes of 44 patients were recorded. This study was approved by the Institutional Review Board of the National Hospital for Tropical Diseases, Hanoi, Vietnam. The need for participant consent was waived by the Ethics Committee of the National Hospital for Tropical Diseases (Decision No.02A/HDDD-NDTU issued on March 30 2020).

### Data analysis

#### Consensus assembly and variant detection

A quality control, assembly and variant calling workflow for Illumina data with the Nextera XT library preparation kit was used [22]. The workflow performed on CLC genomics workbench version 20.0.3 included raw reads firstly trimmed based on read quality and to remove adapters. Trimmed reads were mapped to a data set of eight SARS-CoV-2 genomes from Vietnam in March 2020 submitted to GISAID (https://www.gisaid.org/). This resulted in reads specific to SARS-CoV-2 and non-SARS-CoV-2 reads which primarily mapped to the human reference genome (GCA_000001405.15_GRCh38). Viral reads were mapped to the Wuhan SARS-CoV-2 reference genome (NC_045512) producing a consensus sequence and calling sequence variants of 44 viral isolates. The workflow finally created reports (tables or graphic files) and a track list to summarise results of all steps. Based on variant/amino acid change tables, we filtered true mutations which met a coverage of >30x and greater than 70% of total reads carrying the modification. Novel variants and deletions were confirmed by Sanger sequencing.

The viral consensus sequences were submitted to the GLUE website (http://cov-glue.cvr.gla.ac.uk) to confirm whether any mutations were novel compared to the GISAID database. We combined the mutation report from GLUE with CLC genomics workbench to remove modifications that did not satisfy the threshold for mutation selection. QualiMap application [23] was used to evaluate sequencing depth across the reference genome and calculate mean coverage values.

#### Phylogenetic analysis of study sequences in global context

The study sequences were genotyped using the Pangolin [24] web server (https://pangolin.cog-uk.io/), which revealed that all 44 viruses were of the SARS-CoV-2 B lineage. To examine the diversity and potential sources, study sequences were compared to a random selection of 1000 globally circulating B lineage viruses and other Vietnamese sequences (41 sequences including 5 A lineage viruses) available on GISAID with collection dates reflecting the study period between December 1st 2019 and April 15th 2020. All sequences were first aligned against the prototype strain Wuhan-Hu-1/2019 (GenBank accession NC_045512) with MAFFT [25] before sequences with excessive ambiguities (>400 sites) and divergence (>25 SNPs from prototype) were removed along with those without a specific day of sample collection. The refined alignment was then analysed phylogenetically using a maximum likelihood approach in RAxML [26] with the GTR+G substitution model and 1000 bootstrap replicates. The final tree was visualised and annotated with FigTree [27]. Cluster identification was based on the phylogenetic analysis showing monophyletic clades with a bootstrap value greater than 70.

#### Metadata analysis

Metadata distributed in range and continuously, and their mean and standard deviation (SD) values were reported. Other categorical variables were counted and percentage given per total sample. Microsoft Excel was used to statistically analyze these data.

## Results

### Demographic and epidemiological features of patients with COVID-19 in Vietnam

This study enrolled 44 patients who were selected for inclusion based on a positive SARS-CoV-2 real-time RT PCR result with Ct values of *E* and *RdRp* gene <30. The median age of all patients was 37 years (range 15–74), with the 20–39 years age group the most prevalent, accounting for 20 cases (Table 1). Thirty-two patients were of Vietnamese nationality, and the imported cases had travelled from United Kingdom (n = 15), Russia (n = 6), Germany (n = 5), France (n = 4), Italy (n = 2), Spain (n = 2), Netherlands (n = 1) and countries in Asia (n = 3). The male:female ratio was 23:21.

**Table 1:** Demographic and epidemiological characteristics of laboratory-confirmed COVID-19 cases in Vietnam.

	Cases (44)		Cases (44)
	n (%)		n (%)
**Gender**		**Source of transmission**	
Male	23 (52)	Contact with COVID-19 case in Vietnam	12 (27)
Female	21 (48)	International travel in past 14 days	25 (57)
**Age**		Both	5 (11)
Mean (SD)	37.36 (16.82)	Unknown	2 (5)
Range	15–74	**International travel from country**	
0–9	0	China	0
10–19	6 (14)	India	1 (2)
20–39	20 (45)	Singapore (transit)	1 (2)
40–59	12 (27)	Japan	1 (2)
60–79	6 (14)	UK	15 (34)
≥80	0	France	4 (9)
**Nationality**		Germany	5 (11)
Vietnamese	32 (72)	Italy	2 (5)
UK	7 (16)	Russia	6 (13)
French	3 (7)	Spain	2 (5)
German	2 (5)	Netherlands	1 (2)

In terms of epidemiology, the majority of patients were imported cases (25/44) entering Vietnam from March to April 2020. These cases included returning travellers from Europe, especially the UK. There were no cases from China. While most imported cases (n = 17) were quarantined immediately, 8 cases had contact with relatives and community before SARS-CoV-2 detection and isolation at the NHTD. There were 12 cases infected following contact with positive cases in Vietnam, another five cases were acquired from either international sources or other known contacts, whereas for two cases contact details were unavailable (Table 1).

### Clinical features and treatment

According to the WHO definition [28], we identified symptomatic cases as laboratory-confirmed people displaying any symptoms or signs of respiratory viral infection at admission. Laboratory-confirmed cases not having any symptoms at diagnosis and at 14 day follow-up were classified as asymptomatic. Pre-symptomatic cases were defined as a laboratory-confirmed case who did not have symptoms at diagnosis but developed clinical symptoms within 14 days. Forty-one cases were symptomatic with dry cough (n = 36), fever (n = 20), sore throat (n = 14), diarrhoea (n = 12); there were a few cases with headache, difficulty breathing, muscle aches and fatigue (Table 2). Most patients did not have underlying chronic medical diseases, but 10 had co-morbidities including cancer, diabetes, asthma, hypertension, chronic kidney disease, cerebrovascular disease, gout, vestibular disorder, heart failure or gastroesophageal reflux.

**Table 2:** Clinical characteristics and treatment of 44 patients with COVID-19 admitted to the National Hospital for Tropical Diseases.

	Cases (44)		Cases (44)
	n (%)		n (%)
**Clinical features and history**			
		**Comorbidities**	
Symptomatic	41 (93)	Asthma	1 (2)
Asymptomatic	3 (7)	Diabetes	3 (7)
**Symptoms**		Hypertension	3 (7)
Fever	20 (45)	Cancer	2 (5)
Dry cough	36 (82)	Chronic kidney disease	1 (2)
Sputum production	10 (23)	Cerebrovascular disease	1 (2)
Headache	5 (11)	Other	6 (14)
Nasal congestion	5 (11)	**Imaging**	
Sore throat	14 (32)	Chest X-ray abnormalities	30 (68)
Fatigue	10 (23)	Chest CT abnormalities	34 (77)
Shortness of breath	6 (14)	**Complications**	
Diarrhoea	12 (27)	Acute respiratory distress syndrome	5 (11)
Myalgia	6 (14)	Pneumonia	34 (77)
Other	2 (5)	Other	2 (5)
**Course of Treatment**			
**Hospital stay (days)**		**Treatment received**	
Mean (SD)	25.34 (14.95)	Intravenous antibiotics	11 (25)
<10	5 (11)	Antifungal medications	3 (7)
10–19	10 (23)	Systemic glucocorticoids	3 (7)
20–29	17 (39)	Oxygen therapy without mechanical ventilation	6 (14)
30–39	8 (18)	Invasive ventilation (intubation)	4 (9)
40–49	1 (2)	Extracorporeal membrane oxygenation	1 (2)
50–59	1 (2)	Renal dialysis	2 (5)
60–69	0	Intravenous Immunoglobulin	5 (11)
70–79	2 (5)		
> = 80	0		

There were 34 cases diagnosed with pneumonia based on chest X-ray or CT abnormalities (Table 2): one case had an asymptomatic pneumonia. Notably, four patients developed critical illness with respiratory failure requiring initial oxygen supplementation, invasive intubation (four cases, 9%), with one switched to extracorporeal membrane oxygenation (Table 2 and S1 Table). Only one of the four critically ill patients did not have any underlying chronic disease (S1 Table). Critically ill patients were moved to the intensive care unit (ICU), and transferred to another department when recovering.

In terms of the treatment course, 11 patients were treated with antibiotics, and three of the ICU patients received antifungals and glucocorticoids. Apart from the four critical cases, two patients required non-mechanical oxygenation. Two patients needed renal dialysis, one of whom had a history of chronic kidney disease. Intravenous immunoglobulin (IVIG) was given to five patients (Table 2). Treatment duration was around 25 days, with 17 cases between 20–29 days. Five cases needed under 10 days to recover, while two cases stayed in hospital for up to 79 days. All patients recovered and were discharged home.

### Assembly and phylogenetic analysis of SARS-CoV-2 sequences

**Fig 1:**
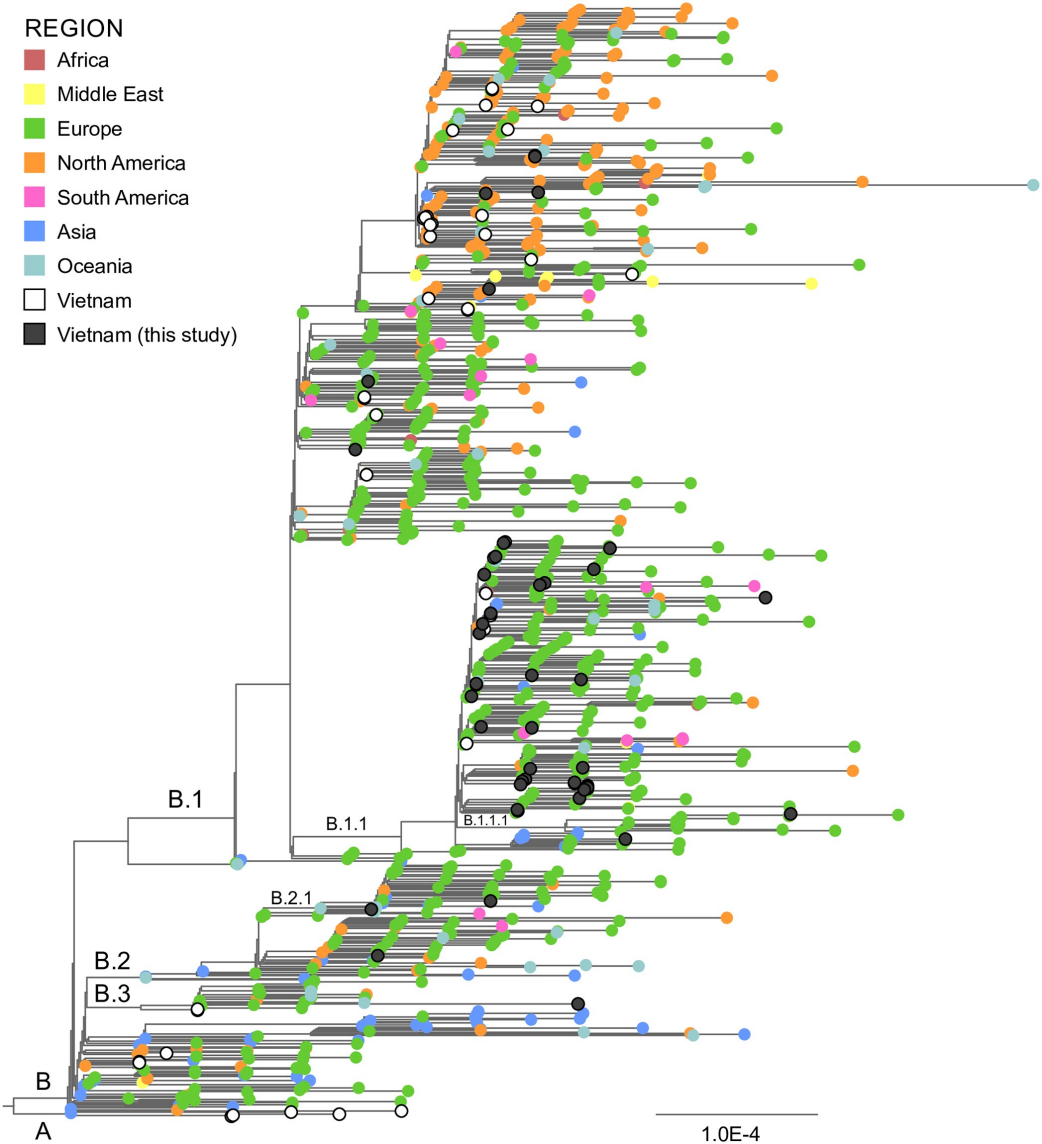
Phylogenetic analysis of SARS-CoV-2 genomes in Vietnam. An alignment of 1056 SARS-CoV-2 sequences including 44 from this study and 41 other Vietnamese sequences was examined using a maximum likelihood approach. Sequences have been coloured according to the provided key and relevant SARS-CoV-2 lineages are indicated. The scale represents the number of substitutions per site.

Forty-four complete SARS-CoV-2 sequences were produced using an amplicon-based approach. For each sequence library, 92–100% of paired end reads mapped to the reference genome sequence (NC_045512). Lengths varied between 29,777 and 29,872 base pairs (bp) at sequencing depths (average coverage across the whole reference genome) greater than 2000x (Table 3). All sequences were submitted to GISAID (https://www.gisaid.org/) with accession numbers listed in S2 Table. When compared to all GISAID sequences, our sequences fell into six lineages including B.1, B.1.1, B.1.1.1, B.2, B.2.1 and B.3, with the dominant lineage being B.1.1 (32/44) (Fig 1). Among the 32 B.1.1 viruses, eleven were isolated from patients who were contacts of positive cases in the community, four were from patients reporting both international travel (from UK and Germany) and contact with positive cases, 16 were from imported cases (UK six cases, Germany two, Russia three, and one case each from Italy, France, Netherlands, Spain and Japan). Two cases with unknown transmission source had B.1 and B.1.1 lineage sequences (S3 Table).

**Table 3:** Genome assembly of 44 SARS-CoV-2 sequences in Vietnam.

Patient ID	Sample ID	Genome assembly			Coverage (mean)
		Total reads	Reads mapped to reference (%)	Sequence length (bp)	
BN1	VNHN_0148	683,040	99.10	29777	8041x
BN2	VNHN_0022	821,174	99.43	29857	4000x
BN3	VNHN_0026	1,654,830	99.80	29824	8200x
BN4	VNHN_0207	1,278,944	96.40	29823	4644x
BN5	VNHN_0301	2,923,360	99.63	29806	14205x
BN6	VNHN_0300	686,522	99.80	29830	3400x
BN7	VNHN_0302	1,427,074	99.57	29826	7018x
BN8	VNHN_0299	1,634,462	99.84	29828	8041x
BN9	VNHN_0418	809,044	98.21	29861	3111x
BN10	VNHN_0419	698,912	99.09	29847	2786x
BN11	VNHN_0554	1,029,724	95.29	29858	3716x
BN12	VNHN_0764	1,905,202	99.81	29823	9362x
BN13	VNHN_0762	2,000,168	99.77	29829	9823x
BN14	VNHN_0847	1,221,394	98.60	29856	5950x
BN15	VNHN_0837	1,209,456	91.86	29824	4227x
BN16	VNHN_0899	861,518	97.83	29821	3148x
BN17	VNHN_1072	763,972	98.16	29844	2824x
BN18	VNHN_1097	754,574	98.81	29826	2999x
BN19	VNHN_0985	1,590,204	99.40	29827	7800x
BN20	VNHN_0897	656,046	99.35	29845	2604x
BN21	VNHN_1098	1,781,052	99.83	29821	8778x
BN22	VNHN_1099	670,404	99.81	29823	3320x
BN23	VNHN_0979	633,110	99.87	29808	3081x
BN24	VNHN_1166	988,242	97.73	29820	3621x
BN25	VNHN_1167	505,349	99.64	29821	2491x
BN26	VNHN_1528	703,289	99.91	29848	3448x
BN27	VNHN_1226	696,214	99.90	29823	3422x
BN28	VNHN_1492	597,053	99.89	29822	3654x
BN29	VNHN_2406	597,240	99.95	29832	2937x
BN30	VNHN_1713	1,517,254	96.84	29824	5515x
BN31	VNHN_1863	538,022	99.95	29826	2660x
BN32	VNHN_3096	625,692	99.87	29842	3031x
BN33	VNHN_3085	436,926	99.93	29820	2162x
BN34	VNHN_3916	432,002	99.96	29824	2153x
BN35	VNHN_3913	640,569	99.69	29822	3156x
BN36	VNHN_3629	596,636	99.96	29823	2952x
BN37	VNHN_4189	579,387	99.96	29826	2862x
BN38	VNHN_4864	697,294	99.98	29826	3458x
BN39	VNHN_4958	920,723	99.82	29872	4532x
BN40	VNHN_4851	766,584	99.94	29828	3794x
BN41	VNHN_4868	536,945	99.94	29824	2639x
BN42	VNHN_4806	769,137	99.86	29855	3776x
BN43	VNHN_4875	681,670	99.30	29825	2822x
BN44	VNHN_5152	708,090	97.31	29819	2587x

Although our sequences were mostly from B.1 viruses, and particularly the B.1.1 lineage, the sequences were relatively dispersed across the phylogeny. There were two notable clusters observed within the B.1.1 lineage: cluster 1 included three samples (VNHN_5152, VNHN_4875, VNHN_4868) and cluster 2 consisted of five samples (VNHN_3085, VNHN_3913, VNHN_3916, VNHN_4864, VNHN_4958) (S1 Fig). Cluster 1 sequences differed from each other at one SNP or one deletion of three nucleotides, while four sequences in cluster 2 were identical and another sequence had a 6-nucleotide deletion compared to others. Interestingly, all samples in cluster 1 were isolated from locally transmitted cases in a local hotspot in Vietnam, and included members of the same family and a neighbour. Cases in cluster 2 were residents returning from different countries: three of them were on the same flight from Germany, one from Japan and the other from Spain. A comparison of our sequences to 41 other Vietnamese sequences on GISAID collected between January and April 2020 showed little if any grouping suggesting most were unique importation events and there was little, if any, mixing between the northern (this study) and southern parts of Vietnam (S1 Fig).

### Mutations in SARS-CoV-2 genomes

Using CLC genomics workbench, we identified 285 mutations covering 67 variant types among the 44 SARS-CoV-2 genomes when compared to the reference genome, averaging 6.5 variants per genome. Sixty-one variants were single nucleotide substitutions, causing 36 non-synonymous and 25 synonymous amino acid changes (Table 4). The most ubiquitous modifications were C3037T, C14408T (P323L) and A23403G (D614G) occurring in 40/44 samples. Two other variants C241T and GGG to AAC at 28881–3 were detected in 39 and 33 sequences, respectively. These variants are key markers to define lineages B.1 (C241T, C3037T, A23403G) and B.1.1 (C241T, C3037T, A23403G, GGG28881..28883AAC), the major lineages in our study. The most common variants were detected in viral sequences amplified from patients with both mild disease and in the ICU.

**Table 4:** Mutations in SARS-CoV-2 genomes and their detection in the GISAID database: Nucleotide variants and amino acid changes.

Location	Mutation type	Reference	Allele	Amino acid change	Coding region	Mutation in database	n (%)
174	SNV	G	A	-			1 (0.35)
241	SNV	C	T	-			39 (13.68)
313	SNV	C	T	-			1 (0.35)
370	SNV	G	A	-			1 (0.35)
508–522	Deletion	TGGTCATGTTATGGT	-	Gly82_Val86del	nsp1	known	2 (0.7)
516–518	Deletion	TTA	-	Met85del	nsp1	known	1 (0.35)
1059	SNV	C	T	T85I	nsp2	known	4 (1.4)
1440	SNV	G	A	G212D	nsp2	known	1 (0.35)
1666	SNV	T	C	-			2 (0.7)
2480	SNV	A	G	I559V	nsp2	known	1 (0.35)
2558	SNV	C	T	P585S	nsp2	known	2 (0.7)
2891	SNV	G	A	A58T	nsp3	known	1 (0.35)
3037	SNV	C	T	-			40 (14.04)
3370	SNV	T	A	-			1 (0.35)
4002	SNV	C	T	T428I	nsp3	known	1 (0.35)
4655	SNV	C	T	R646W	nsp3	known	1 (0.35)
4733	SNV	C	T	L672F	nsp3	novel	1 (0.35)
4908	SNV	G	A	G730D	nsp3	known	1 (0.35)
5513	SNV	G	A	-			1 (0.35)
5514	Deletion	T	-	Frameshift	nsp3	novel	1 (0.35)
6027	SNV	C	T	P1103L	nsp3	known	1 (0.35)
6276	SNV	A	G	K1186R	nsp3	known	1 (0.35)
6502	SNV	A	G	-			1 (0.35)
8422	SNV	G	A	M1901I	nsp3	known	1 (0.35)
9172	SNV	T	C	-			1 (0.35)
9983	SNV	G	A	D477N	nsp4	known	1 (0.35)
10054	SNV	G	A	-			1 (0.35)
10097	SNV	G	A	G15S	nsp5	known	1 (0.35)
10948	SNV	A	G	-			1 (0.35)
11083	SNV	G	T	L37F	nsp6	known	3 (1.05)
11824	SNV	C	T	-			3 (1.05)
13536	SNV	C	T	-			1 (0.35)
13922	SNV	A	T	D161V	nsp12	known	1 (0.35)
14184	SNV	C	T	-			2 (0.7)
14408	SNV	C	T	P323L	nsp12	known	40 (14.04)
14452	SNV	G	T	V338F	nsp12	known	1 (0.35)
14793	SNV	C	T	-			1 (0.35)
14805	SNV	C	T	-			3 (1.05)
15720	SNV	C	T	-			1 (0.35)
18021	SNV	G	T	R595S	nsp13	known	1 (0.35)
18877	SNV	C	T	-			1 (0.35)
19839	SNV	T	C	-			6 (2.11)
20578	SNV	G	T	V320L	nsp15	known	1 (0.35)
21057	SNV	C	T	-			2 (0.7)
21058	SNV	C	T	P134S	nsp16	known	1 (0.35)
21077	SNV	C	T	T140I	nsp16	known	1 (0.35)
21724	SNV	G	T	L54F	S	known	2 (0.7)
22323	SNV	C	T	S254F	S	known	1 (0.35)
23403	SNV	A	G	D614G	S	known	40 (14.04)
23731	SNV	C	T	-			1 (0.35)
25311	SNV	G	T	C1250F	S	known	1 (0.35)
25563	SNV	G	T	Q57H	ORF 3a	known	5 (1.75)
26028	SNV	C	T	-			1 (0.35)
26144	SNV	G	T	G251V	ORF 3a	known	3 (1.05)
26256	SNV	C	T	-			1 (0.35)
26530	SNV	A	G	D3G	M	known	1 (0.35)
26730	SNV	G	T	V70F	M	known	1 (0.35)
27635	SNV	C	T	S81L	ORF 7a	known	1 (0.35)
27679	SNV	C	T	L96F	ORF 7a	known	2 (0.7)
27874	SNV	C	T	T40I	ORF 7b	known	1 (0.35)
27695–27700	Deletion	TTCTTA	-	L102_I103del	ORF 7a	known	1 (0.35)
27792–27793	Deletion	TT	-	Frameshift	ORF 7b	novel	1 (0.35)
28881–28883	MNV	GGG	AAC	R203K; G204R	N	known	33 (11.58)
28812	SNV	G	T	S180I	N	known	1 (0.35)
28905	SNV	C	T	A211V	N	known	5 (1.75)
29122	SNV	A	C	Q283H	N	novel	1 (0.35)
29546	SNV	C	A	-			1 (0.35)
						**Total**	**285**

SNV: single nucleotide variant, MNV: Multiple nucleotide variant, nsp: non-structural protein, OFR: open reading frame.

There were three deletions of 15, six and three nucleotides, resulting in loss of five, two and one amino acids respectively, while two deletions of one or two nucleotides led to frameshifts. No insertions among the 44 genome sequences were detected. Two novel mutations were associated with frameshifts as mentioned previously, the others were non-synonymous changes, namely C4733T (L672F) and A29122C (Q283H). Novel mutations occurred in non-structural protein 3 (ORF 1a), open reading frame 7b and N coding regions on SARS-CoV-2 genome, and were detected in three different genomes (VNHN_0148, VNHN_0762, VNHN_1166). These mutations were not associated with differences in phenotype of illness, although numbers were small.

There were 14 ORFs across the SARS-CoV-2 genome [4]. Mutations detected in our study often occurred in the nsp3 region (ORF1ab) with nine variant types, followed by N, S and nsp2 regions with four variant types (Table 4). The conservative coding regions without any modifications were E, ORF6, ORF8 and ORF10. Notably, four types of mutations detected in the Spike gene did not occur in the receptor binding domain (RBD: residue 319–541) that binds to the human receptor hACE2 for viral entry [3].

## Discussion

NHTD is a front-line national hospital in Hanoi for isolation and treatment of laboratory-confirmed COVID-19 cases for the northern part of Vietnam. Forty-four SARS-CoV-2 RT-PCR patients with Ct values <30 had SARS-CoV-2 WGS performed with the aim of characterising lineages and identifying mutations in patients with different transmission sources in Vietnam. This study also describes the clinical and epidemiological features, treatment and outcomes of 44 COVID-19 patients.

The ages, gender and origins of COVID-19 disease are consistent with a Vietnamese Ministry of Health report summarising the demographic and epidemiological data of 207 COVID-19 cases in Vietnam from January 23 to May 1 2020 [5]. Among our 44 patients, 41 had respiratory symptoms on admission. In a study in southern Vietnam conducted in a quarantine facility rather than a referral hospital, only 57% of patients reported symptoms [18]. This difference may be due to characteristics of patients varying between a national hospital and government quarantine centres, or due to sample selection: we examined patients with Ct <30 in the real-time RT PCR, thus possibly not representing all cases at the NHTD. Common symptoms such as dry cough and fever reported in our study are similar to earlier reports of COVID-19 in China [29, 30] and Vietnam [7, 9, 16]. However, most patients did not have underlying chronic diseases, and most (77%) had pneumonia with abnormalities on chest imaging, including one asymptomatic case. The median hospital stay was 25 days, which was longer than that of other reports from Vietnam of either 14–19 or 15–18 days [8, 13].

The SARS-CoV-2 genomes in our study are complete, high coverage sequences, accounting for more than 50% of available Vietnamese genomes of SARS-CoV-2 submitted to GISAID to date. Our phylogenetic analysis of 85 SARS-CoV-2 sequences from Vietnam collected up to April 15th 2020 submitted to GISAID showed almost all were B.1 lineage and sublineages. Most of our viral genomes belonged to the B.1.1 lineage, the dominant lineage in the UK as updated on May 19 2020 [31]. This supports the epidemiological data where 50% (15/30) of international travellers in this study had returned from UK. We identified two significant clusters of B.1.1 lineage, each containing three and five samples. Combined with epidemiological factors, the cluster of three samples originated from a local hotspot village whereas the cluster of five cases was from three different countries. There was a significant difference between viruses sampled in south and north Vietnam, mainly B.1 versus B.1.1 respectively (S1 Fig). In addition, viruses within each region in Vietnam did not cluster together but with sequences from other countries, suggesting multiple introduction events and limited local transmission.

We characterised the three most prevalent variants detected in 40/44 completed genome sequences, namely C3037T (synonymous substitution) and two non-synonymous mutations C14408T (nsp12: P323L), A23403G (S: D614G). Another study analysed over 10,000 SARS-CoV-2 genomes from 68 countries from four databases showed an identical result, with over 6000 sequences carrying these three variants [32]. Interestingly, these three variants always occurred together, and sometimes they were accompanied by a synonymous mutation C241T (in 39 genomes) or multiple nucleotide substitutions GGG28881..28883AAC (N: R203K, G204R; in 33 genomes), suggesting linkage of mutations among these strains. The prevalence of these mutations was consistent with major lineages identified in this study. The linkage of four changes (C241T, C3037T, C14408T, A23403T) has been described in a comprehensive study of Spike protein variants especially related to the D614G transition [33]. The D614G mutation was first detected in a SARS-CoV-2 sequence from Germany in January 2020 [34]. Subsequently, haplotypes combining these four variants appeared in Italy in February 2020, then spread throughout Europe and currently account for 78% of worldwide sequences submitted to GISAID (May 29 2020), forming the G614 SARS-CoV-2 strain [33]. Of the SARS-CoV-2 genomes from the first two patients in Vietnam (one from Wuhan who transmitted to a second case), the D614G change was not found [9]. The B.1 lineage and its sublineages have formed the majority of sequences from Vietnam since March 2020 (S1 Fig) and harbour D614G. The current variants detected in our study together with other genome sequences indicate a similar trend of transition of D614 to G614 in Vietnam, which confirms that the main transmission source of COVID-19 here was from Europe rather than from China (where the progenitor D614 strains predominate). The study by Kyogama et al. [32] agreed with our finding that nsp3 protein (ORF1ab) most usually occurred as mis-sense variants. We did not find mutations in the E, ORF6, ORF8 and ORF10 coding regions. We identified other mutations on the Spike protein (L54F, S254F, C1250F), however these changes did not occur in the receptor binding domain. Four new unique variants were detected in three SARS-CoV-2 genomes in our study, including C4733T (nsp3: L672F), A29122C (N: Q283H), deletion TT 27792..27793 (OFR7b: Leu14 frameshift) and deletion T 5514 (nsp3: Val931 frameshift). However, we did not find a link between these new mutations and disease severity.

This study provides an insight into the clinical features of COVID-19 and evolutionary trends of SARS-CoV-2 in Vietnam. Genomic surveillance combined with field epidemiology will remain crucial to track and trace transmission.

## Supporting information

S1 Fig Phylogenetic analysis of SARS-CoV-2 genomes in Vietnam with taxa labels. An alignment of 1056 SARS-CoV-2 sequences including 44 from this study and 41 other Vietnamese sequences was examined using a maximum likelihood approach. This corresponds to the same phylogeny shown in Fig 1, except it has been expanded to show individual taxa labels. Sequences have been coloured according to the provided key and SARS-CoV-2 lineages and bootstrap support values are shown for all branches. The scale represents the number of substitutions per site.
(TIF)

S1 Table Clinical characteristics and treatment of critically ill COVID-19 patients. (DOCX)

S2 Table Accession number of Vietnamese SARS-CoV-2 genome sequences submitted to GISAID. (DOCX)

S3 Table Lineage of SARS-CoV-2 strains collected at the National Hospital for Tropical Diseases. (DOCX)
