# Supplementary figures and images for: Genetic diversity of SARS-CoV-2 and clinical, epidemiological characteristics of COVID-19 patients in Hanoi, Vietnam
Source: PLoS One. 2020 Nov 17;15(11):e0242537. doi: 10.1371/journal.pone.0242537 (PMC7671498; doi:10.1371/journal.pone.0242537)

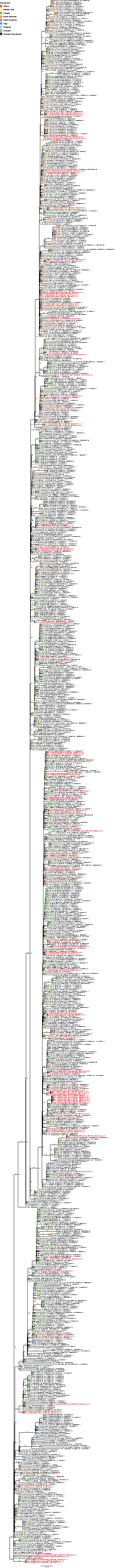

Supplement: S1 Fig — An alignment of 1056 SARS-CoV-2 sequences including 44 from this study and 41 other Vietnamese sequences was examined using a maximum likelihood approach. This corresponds to the same phylogeny shown in Fig 1, except it has been expanded to show individual taxa labels. Sequences have been coloured according to the provided key and SARS-CoV-2 lineages and bootstrap support values are shown for all branches. The scale represents the number of substitutions per site. (TIF) [file pone.0242537.s001.tif]
