# Supplementary material for: Genetic diversity of SARS-CoV-2 and clinical, epidemiological characteristics of COVID-19 patients in Hanoi, Vietnam
Source: PLoS One. 2020 Nov 17;15(11):e0242537. doi: 10.1371/journal.pone.0242537 (PMC7671498; doi:10.1371/journal.pone.0242537)
Supplement: S1 Table — (DOCX) [file pone.0242537.s002.docx]

**S1 Table. Clinical characteristics and treatment of critically ill COVID-19 patients**

| **Patient ID** | **BN3** | **BN6** | **BN9** | **BN12** |
| --- | --- | --- | --- | --- |
| **Sample ID** | VNHN_0026 | VNHN_0300 | VNHN_0418 | VNHN_0764 |
| **Treatment duration (days)** | 73 | 21 | 22 | 55 |
| **Symptoms at admission** |  |  |  |  |
| Fever | Y | Y | Y | Y |
| Dry cough | Y | Y | Y | Y |
| Sputum production | Y | N | Y | N |
| Headache | Y | N | Y | N |
| Nasal congestion | N | N | Y | N |
| Sore throat | Y | N | Y | N |
| Fatigue | Y | N | Y | Y |
| Shortness of breath | Y | N | Y | Y |
| Diarrhoea | Y | N | N | Y |
| Myalgia | Y | Y | N | N |
| **Coexisting diseases** |  |  |  |  |
| Asthma | N | N | N | N |
| Diabetes | N | N | Y | N |
| Hypertension | N | N | Y | N |
| Cancer | N | Y | N | N |
| Chronic kidney diseases | N | N | N | N |
| Cerebrovascular diseases | N | N | Y | N |
| Vestibular disorder | Y | N | N | N |
| **Chest Imaging** |  |  |  |  |
| X-ray abnormalities | Y | Y | Y | Y |
| CT abnormalities | Y | Y | Y | Y |
| **Complications** |  |  |  |  |
| Acute respiratory distress syndrome | Y | Y | Y | Y |
| Pneumonia | Y | Y | Y | Y |
| Other (nosocomial infection, acute liver failure) | N | N | Y | N |
| **Oxygen supplementation** |  |  |  |  |
| Non-mechanical oxygen therapy | Y | Y | Y | Y |
| Invasive ventilation | Y | Y | Y | Y |
| Extracorporeal membrane oxygenation | Y | N | N | N |
| **Treatment** |  |  |  |  |
| Intravenous antibiotics | Y | Y | Y | Y |
| Antifungal medication | Y | Y | Y | N |
| Glucocorticoids | Y | N | Y | Y |
| Renal dialysis | N | N | Y | N |
| Intravenous Immunoglobulin | Y | Y | Y | Y |
