# Supplementary material for: Genetic diversity of SARS-CoV-2 and clinical, epidemiological characteristics of COVID-19 patients in Hanoi, Vietnam
Source: PLoS One. 2020 Nov 17;15(11):e0242537. doi: 10.1371/journal.pone.0242537 (PMC7671498; doi:10.1371/journal.pone.0242537)
Supplement: S2 Table — (DOCX) [file pone.0242537.s003.docx]

**S2 Table. Accession number of Vietnamese SARS-CoV-2 genome sequences submitted to GISAID**

| **No.** | **Virus name** | **Accession ID** | **Collection date** |
| --- | --- | --- | --- |
| 1 | hCoV-19/Vietnam/VNHN_0022/2020 | EPI_ISL_435303 | 07/03/2020 |
| 2 | hCoV-19/Vietnam/VNHN_0026/2020 | EPI_ISL_435305 | 07/03/2020 |
| 3 | hCoV-19/Vietnam/VNHN_0299/2020 | EPI_ISL_435308 | 09/03/2020 |
| 4 | hCoV-19/Vietnam/VNHN_0300/2020 | EPI_ISL_435310 | 09/03/2020 |
| 5 | hCoV-19/Vietnam/VNHN_0418/2020 | EPI_ISL_435311 | 10/03/2020 |
| 6 | hCoV-19/Vietnam/VNHN_0419/2020 | EPI_ISL_435312 | 10/03/2020 |
| 7 | hCoV-19/Vietnam/VNHN_0762/2020 | EPI_ISL_435313 | 13/03/2020 |
| 8 | hCoV-19/Vietnam/VNHN_0764/2020 | EPI_ISL_435314 | 13/03/2020 |
| 9 | hCoV-19/Vietnam/VNHN_0837/2020 | EPI_ISL_435315 | 15/03/2020 |
| 10 | hCoV-19/Vietnam/VNHN_0985/2020 | EPI_ISL_435316 | 17/03/2020 |
| 11 | hCoV-19/Vietnam/VNHN_1097/2020 | EPI_ISL_435317 | 18/03/2020 |
| 12 | hCoV-19/Vietnam/VNHN_1098/2020 | EPI_ISL_455694 | 18/03/2020 |
| 13 | hCoV-19/Vietnam/VNHN_1099/2020 | EPI_ISL_455695 | 18/03/2020 |
| 14 | hCoV-19/Vietnam/VNHN_1167/2020 | EPI_ISL_455696 | 18/03/2020 |
| 15 | hCoV-19/Vietnam/VNHN_1528/2020 | EPI_ISL_455697 | 22/03/2020 |
| 16 | hCoV-19/Vietnam/VNHN_1226/2020 | EPI_ISL_455698 | 19/03/2020 |
| 17 | hCoV-19/Vietnam/VNHN_1492/2020 | EPI_ISL_455699 | 21/03/2020 |
| 18 | hCoV-19/Vietnam/VNHN_2406/2020 | EPI_ISL_455700 | 25/03/2020 |
| 19 | hCoV-19/Vietnam/VNHN_1713/2020 | EPI_ISL_455701 | 23/03/2020 |
| 20 | hCoV-19/Vietnam/VNHN_3096/2020 | EPI_ISL_455702 | 27/03/2020 |
| 21 | hCoV-19/Vietnam/VNHN_3085/2020 | EPI_ISL_455703 | 27/03/2020 |
| 22 | hCoV-19/Vietnam/VNHN_3916/2020 | EPI_ISL_455704 | 31/03/2020 |
| 23 | hCoV-19/Vietnam/VNHN_3913/2020 | EPI_ISL_455705 | 31/03/2020 |
| 24 | hCoV-19/Vietnam/VNHN_3629/2020 | EPI_ISL_455706 | 30/03/2020 |
| 25 | hCoV-19/Vietnam/VNHN_4189/2020 | EPI_ISL_455707 | 02/04/2020 |
| 26 | hCoV-19/Vietnam/VNHN_4864/2020 | EPI_ISL_455708 | 10/04/2020 |
| 27 | hCoV-19/Vietnam/VNHN_4851/2020 | EPI_ISL_455709 | 09/04/2020 |
| 28 | hCoV-19/Vietnam/VNHN_4868/2020 | EPI_ISL_455710 | 09/04/2020 |
| 29 | hCoV-19/Vietnam/VNHN_4806/2020 | EPI_ISL_455711 | 08/04/2020 |
| 30 | hCoV-19/Vietnam/VNHN_4875/2020 | EPI_ISL_455712 | 10/04/2020 |
| 31 | hCoV-19/Vietnam/VNHN_0148/2020 | EPI_ISL_455713 | 06/03/2020 |
| 32 | hCoV-19/Vietnam/VNHN_0301/2020 | EPI_ISL_455714 | 09/03/2020 |
| 33 | hCoV-19/Vietnam/VNHN_0302/2020 | EPI_ISL_455715 | 09/03/2020 |
| 34 | hCoV-19/Vietnam/VNHN_0979/2020 | EPI_ISL_455716 | 17/03/2020 |
| 35 | hCoV-19/Vietnam/VNHN_1072/2020 | EPI_ISL_455717 | 17/03/2020 |
| 36 | hCoV-19/Vietnam/VNHN_4958/2020 | EPI_ISL_455718 | 10/04/2020 |
| 37 | hCoV-19/Vietnam/VNHN_0207/2020 | EPI_ISL_511891 | 08/03/2020 |
| 38 | hCoV-19/Vietnam/VNHN_0554/2020 | EPI_ISL_511892 | 11/03/2020 |
| 39 | hCoV-19/Vietnam/VNHN_0847/2020 | EPI_ISL_511893 | 15/03/2020 |
| 40 | hCoV-19/Vietnam/VNHN_0897/2020 | EPI_ISL_511894 | 16/03/2020 |
| 41 | hCoV-19/Vietnam/VNHN_0899/2020 | EPI_ISL_511895 | 16/03/2020 |
| 42 | hCoV-19/Vietnam/VNHN_1166/2020 | EPI_ISL_511896 | 18/03/2020 |
| 43 | hCoV-19/Vietnam/VNHN_1863/2020 | EPI_ISL_511897 | 23/03/2020 |
| 44 | hCoV-19/Vietnam/VNHN_5152/2020 | EPI_ISL_511898 | 14/04/2020 |
